# The role of overweight and obesity in adverse cardiovascular disease mortality trends: an analysis of multiple cause of death data from Australia and the USA

**DOI:** 10.1186/s12916-020-01666-y

**Published:** 2020-08-04

**Authors:** Tim Adair, Alan D. Lopez

**Affiliations:** Melbourne School of Population and Global Health, The University of Melbourne, Level 5, Building 379, 207 Bouverie Street, Carlton, Victoria 3010 Australia

**Keywords:** Cardiovascular diseases, Mortality, Obesity, Diabetes, Chronic kidney disease, Hypertensive heart disease, Multiple causes of death

## Abstract

**Background:**

In recent years, there have been adverse trends in premature cardiovascular disease (CVD) mortality rates (35–74 years) in the USA and Australia. Following long-term declines, rates in the USA are now increasing while falls in Australia have slowed rapidly. These two countries also have the highest adult obesity prevalence of high-income countries. This study investigates the role of overweight and obesity in their recent CVD mortality trends by using multiple cause of death (MCOD) data—direct individual-level evidence from death certificates—and linking the findings to cohort lifetime obesity prevalence.

**Methods:**

We identified overweight- and obesity-related mortality as any CVD reported on the death certificate (CVD MCOD) with one or more of diabetes, chronic kidney disease, obesity, lipidemias or hypertensive heart disease (DKOLH-CVD), causes strongly associated with overweight and obesity. DKOLH-CVD comprises 50% of US and 40% of Australian CVD MCOD mortality. Trends in premature age-standardized death rates were compared between DKOLH-CVD and other CVD MCOD deaths (non-DKOLH-CVD). Deaths from 2000 to 2017 in the USA and 2006–2016 in Australia were analyzed. Trends in in age-specific DKOLH-CVD death rates were related to cohort relative lifetime obesity prevalence.

**Results:**

Each country’s DKOLH-CVD mortality rate rose by 3% per annum in the most recent year, but previous declines had reversed more rapidly in Australia. Non-DKOLH-CVD mortality in the USA increased in 2017 after declining strongly in the early 2000s, but in Australia it has continued declining in stark contrast to DKOLH-CVD. There were larger increases in DKOLH-CVD mortality rates at successively younger ages, strongly related with higher relative lifetime obesity prevalence in younger cohorts.

**Conclusions:**

The increase in DKOLH-CVD mortality in each country suggests that overweight and obesity has likely been a key driver of the recent slowdown or reversal of CVD mortality decline in both countries. The larger recent increases in DKOLH-CVD mortality and higher lifetime obesity prevalence in younger age groups are very concerning and are likely to adversely impact CVD mortality trends and hence life expectancy in future. MCOD data is a valuable but underutilized source of data to track important mortality trends.

## Background

Recent evidence has revealed that cardiovascular disease (CVD) mortality rates in many high-income countries, after falling by up to 80% over the past four decades, are either declining at progressively slower rates or, in some countries, even increasing [1]. This phenomenon is occurring in countries spanning a wide range of epidemiological environments and levels of CVD mortality. The worsening trends in CVD mortality are most apparent for premature mortality, defined here as a death at ages 35–74 years, with rates increasing in the USA for both sexes, and in Canada for females. On average, the latest annual decline in death rates across high-income countries is about half that observed in the first decade of this century. These trends are particularly concerning given that life expectancy improvements in some high-income countries are slowing or have even stalled and that continuing large declines in CVD, as a leading cause of death, would be a major contributor to future gains in life expectancy [2–5].

These trends in CVD mortality are occurring concurrently with, or soon after, adverse trends in overweight and obesity in many high-income countries. Age-standardized adult obesity prevalence (body mass index (BMI) greater than or equal to 30.0 kg/m^2^) in high-income countries increased from 10% in 1980 to 21% in 2015, reaching as high as one third of adults in the USA [6, 7]. There is strong evidence linking overweight and obesity (BMI greater than or equal to 25.0 kg/m^2^) and all-cause mortality, especially CVD mortality, at least in high-income populations [8–10]. An analysis of 57 prospective studies concluded that obesity significantly increases mortality risks, being comparable to smoking at more severe obesity levels [9]. Globally, the Global Burden of Disease (GBD) Study has estimated that over two thirds of deaths attributed to overweight and obesity occur through CVD [6]. While CVD mortality trends are also influenced by a range of other factors, such as smoking, blood pressure, and cholesterol, as well as the quality of and access to health care, its future direction will undoubtedly be influenced by the prevalence of overweight and obesity [11].

The USA and Australia are the 1st- and 2nd-ranked high-income countries, respectively, for adult obesity prevalence, and are also among the most prominent group of countries where premature CVD mortality is also either increasing (USA) or declining at a rapidly slowing rate (Australia) [7]. In both countries, obesity has increased rapidly in recent decades, and in Australia, obesity rates are significantly higher in progressively younger cohorts, suggesting potentially even worse premature CVD mortality rates in the future [7, 12]. Previous research in the USA has suggested that rising BMI in the USA reduced the rate of all-cause mortality decline between 1988 and 2011 by about one quarter, while another study has forecast that continued rises in obesity will more than negate the positive effects on US life expectancy of smoking declines [4, 13]. A 2005 analysis forecast that rising obesity could potentially result in a decline in US life expectancy, now confirmed by official statistics for recent years [14, 15]. Additionally, in 26 European countries, obesity was estimated to have reduced life expectancy gains between 1975 and 2012 by an average 0.78 years for men and 0.30 years for women [16]. No similar studies have been conducted in Australia, although rising obesity levels has been identified as one of the possible factors underlying the recent slowdown in life expectancy gains [3]. Overall, overweight and obesity are estimated to cause 9% of the total burden of disease and injuries in Australia and 12% in the USA [17].

One approach to assessing the role of overweight and obesity on CVD mortality is by using multiple cause of death (MCOD) data [18]. MCOD data include all causes of death reported on the International Form of Medical Certificate of Cause of Death (MCCD or death certificate) for each registered death. Most commonly, only the underlying cause of death (UCOD) is reported in cause of death statistics, that is, the disease or injury that initiated the train of morbid events that led to the death [19]. For CVD mortality, more often than not occurring as a co-morbid condition, analysis of UCOD data alone can mask important cause contributions that can otherwise be revealed using MCOD data [20]. However, despite their strengths, MCOD data have been underutilized in the analysis of mortality, although interest in exploiting the epidemiological content of such data has risen over the past decade [18].

The use of MCOD data to assess overweight- and obesity-related mortality can be conducted by analyzing specific causes of death that are known to be largely due to this risk factor. There is a substantial body of evidence to suggest that high BMI and especially obesity is associated with a heightened risk of mortality from diabetes, hypertensive heart disease (compared with other CVDs such as ischemic heart disease), and chronic kidney disease, as well as increasing levels of non-HDL cholesterol (the cause of lipidemias) [6, 9, 21, 22]. The GBD Study estimates that, at ages 35–74 years, 56% of deaths in Australia and 63% of deaths in the USA with an underlying cause of death of hypertensive heart disease, diabetes, or chronic kidney disease could be attributed to overweight and obesity (i.e., high BMI) [17]. The corresponding estimates for all CVD mortality were 34% (Australia) and 41% (USA). Furthermore, 67% of all deaths attributed to overweight and obesity at ages 35–74 years in Australia and 80% in the USA have an underlying cause of either diabetes, chronic kidney disease, or CVD (which includes hypertensive heart disease).

Hence, it is insightful and appropriate to analyze overweight- and obesity-related mortality using MCOD data with CVD mortality reported with one or more of hypertensive heart disease, diabetes, or chronic kidney disease on the death certificate. While this combination of causes is not wholly due to overweight and obesity, and does not comprise all overweight and obesity-related mortality, it is likely to be a strong proxy for the contribution of overweight and especially obesity to recent trends in CVD mortality. It could also be argued that such direct individual-level evidence accumulated from death certificates is likely to yield a more reliable estimate of the contribution of overweight and obesity to CVD mortality than the GBD’s (and others’) use of population attributable fractions which are calculated based on high BMI prevalence from sample surveys, relative risks of mortality from high BMI compared with a theoretical minimum population exposure distribution based on review of the literature, and CVD reported as the UCOD [6, 16].

In this study, we use MCOD data in Australia and the USA to assess the role of overweight and obesity in recent trends in CVD mortality. We first examine trends in CVD UCOD and MCOD mortality for all ages and separately for premature mortality (deaths at ages 35–74 years). We then partition premature CVD MCOD mortality into deaths with and without any mention on the death certificate of diabetes, chronic kidney disease, lipidemias, obesity, or hypertensive heart disease, henceforth referred to by the acronyms *DKOLH-CVD* and non-*DKOLH-CVD*. We then link the findings to cohort obesity prevalence.

## Methods

We obtained MCOD data for deaths occurring in Australia between 2006 and 2016 and in the USA between 2000 and 2017. MCOD data for Australia were available from the Australian Cause of Death Unit Record File (COD URF) of all deaths registered in Australia [23]. Deaths are registered by each State and Territory Registry of Births, Deaths and Marriages and the State and Chief Coroners through the National Coronial Information System (NCIS) and compiled and coded by the Australian Bureau of Statistics (ABS) [23]. The COD URF is maintained by the Australian Coordinating Registry (ACR) based at the Queensland Registry of Births, Deaths and Marriages. The COD URF comprises data on deaths registered from 2006 to 2017. However, we only used deaths that occurred in 2006–2016 because in 2017, there was a sharp decline in deaths registered in October–December compared with the earlier months of that year due to a higher number of late registrations than in preceding years. Population data by year, sex, and 5-year age group (up to 95+ years) were obtained from the ABS [24]. The COD URF does not separately identify registered deaths of persons usually resident overseas, so we could not exclude these from the study.

US MCOD data were obtained from the National Center for Health Statistics (NCHS) Multiple Cause of Death Data file [25]. We excluded deaths of foreign residents. US population data by year, sex, and 5-year age group (up to 95+ years) were taken from the Global Burden of Disease Study because official population estimates were not available for specific age groups above 85 years [26, 27].

Both Australia and the USA use the International Form of MCCD, which requires the certifier to report the sequence or chain of events leading to death, with the underlying cause stated on the bottom line in part 1, and to report other significant conditions contributing to death in part 2. In both the Australian and US MCOD datasets, causes of death are reported as International Classification of Diseases Volume 10 (ICD-10) codes (shown in Additional file 1: Table S1) as either “entity axis” or “record axis” conditions. Entity axis data show the ICD-10 codes based on how the causes appeared on the death certificate, including the line number and position number on the certificate. Record axis data show all conditions associated with the death after the application the ICD-10 coding rules, including modification rules and improbable sequence rules [23]. In our study, we used entity axis data because we observed that US chronic kidney disease mentions according to the record axis data declined sharply from 2010 compared with entity axis data; over the same period in Australia, there was little change. However, the choice of entity or record axis data had little impact on the final results, most likely because many of the chronic kidney disease deaths were reclassified within the cluster of overweight- and obesity-related causes (Additional file 2: Table S2 and Additional file 3: Table S3).

We initially analyzed trends in CVD mortality as both a UCOD and a MCOD (i.e., reported anywhere on the death certificate). For CVD MCOD, we excluded cardiac arrest unless it was the UCOD because cardiac arrest could be the immediate cause of death where all other causes are unrelated to CVD [28]. We divided CVD MCOD deaths into two groups according to whether they were associated with overweight and obesity or not: (a) DKOLH-CVD, that is, any mention on the death certificate of diabetes, chronic kidney disease, obesity, lipidemias, or hypertensive heart disease, and (b) non-DKOLH-CVD, i.e., all other CVD MCOD deaths. DKOLH-CVD comprises 50% of US CVD MCOD mortality while 40% of Australian CVD MCOD mortality (Additional file 1: Table S1).

Our approach contrasts with a previous study that analyzed obesity mortality based on the reporting of obesity on the death certificate [18]. That study found that the obesity mortality rate in the USA is similar to that for France and Italy, despite the much higher obesity prevalence in the USA, suggesting that obesity is likely to have been under-reported [18]. This is confirmed by other studies in the USA and Switzerland showing that obesity is under-reported in hospital discharge data, in part because clinical conditions rather than risk factors are preferred when completing medical records [29, 30]. In Australia, hospital data were unable to be used to measure obesity among admitted patients [31]. Hence, reporting of obesity alone is not expected to capture all obesity-related mortality. Despite this, there is no evidence to suggest that other DKOLH-CVD causes are under-reported and, further, our approach’s inclusion of these other causes will likely capture much of obesity-related mortality.

To assess the extent that the DKOLH-CVD causes correlate with each other, when compared with other causes reported within CVD MCOD deaths, we conducted principal components analysis (PCA) in each country for those causes listed in Additional file 1: Table S1. The results in Additional file 4: Table S4 for both Australia and the USA show that, aside from the high correlation of causes that are related with the presence of sepsis within CVD MCOD deaths (e.g., digestive diseases such as vascular disorders of the intestine, paralytic ileus and intestinal obstruction), the component with the next highest eigenvalue has each of the DKOLH-CVD causes as having the five highest scores. That is, the DKOLH-CVD causes are more correlated with each other when compared with other causes, a finding consistent with the epidemiological evidence linking these causes with a common risk factor, namely overweight and obesity.

We analyzed trends in age-standardized death rates for CVD UCOD and CVD MCOD for all ages and the age group 35–74 years. DKOLH-CVD and non-DKOLH-CVD were only analyzed for ages 35–74 years, because it is at these ages where CVD trends and obesity levels were least favorable in each country. Trends in the specific causes within DKOLH-CVD were also assessed. All age-standardized death rates were standardized using the GBD age standard, which enables comparisons with similar analyses in other countries [32]. We also assessed trends in age-specific death rates for 5-year age groups for the USA and, because of a smaller population, 10-year age groups for Australia. All death rates were smoothed over time with local polynomial smoothing using Stata 15.0; annual and annualized changes in death rates were calculated from these smoothed rates [33, 34]. For many of the analyses, the US data were assessed from 2005 to 2017 to be more consistent with the availability of data for Australia from 2006 to 2016.

One potential limitation of analyzing MCOD data is that trends could be biased by an increase in the number of causes reported on the death certificate over time. However, according to the cause list shown in Additional file 1: Table S1, in Australia the average number of causes reported per CVD MCOD increased only slightly, from 2.79 in 2006 to 2.86 in 2016, and by not much more in the USA (2.56 in 2005; 2.78 in 2017). This modest increase in number of conditions reported is highly unlikely to bias results and could well reflect a real increase in the presence of chronic conditions among CVD deaths.

We also compared trends in age-specific DKOLH-CVD death rates with a measure of cohort relative lifetime obesity prevalence to reflect the increased risk of mortality where BMI is high throughout the life course [35]. Using GBD obesity prevalence estimates from 1980 to 2015 by year, sex, and 5-year age groups, we calculated relative obesity prevalence for each 5-year cohort based on age at the most recent year of mortality data. This was conducted by firstly splitting obesity prevalence estimates for 5-year age groups into single years of age using, for each year, a 5th degree polynomial function over age, and then averaging each cohort’s prevalence at every age 5 years and older over the period 1980–2015. We then divided average cohort prevalence by average obesity prevalence for those same ages during 1980–2015. Relative obesity prevalence for each cohort is presented as a percentage above or below the average prevalence for 1980–2015.

## Results

**Fig. 1:**
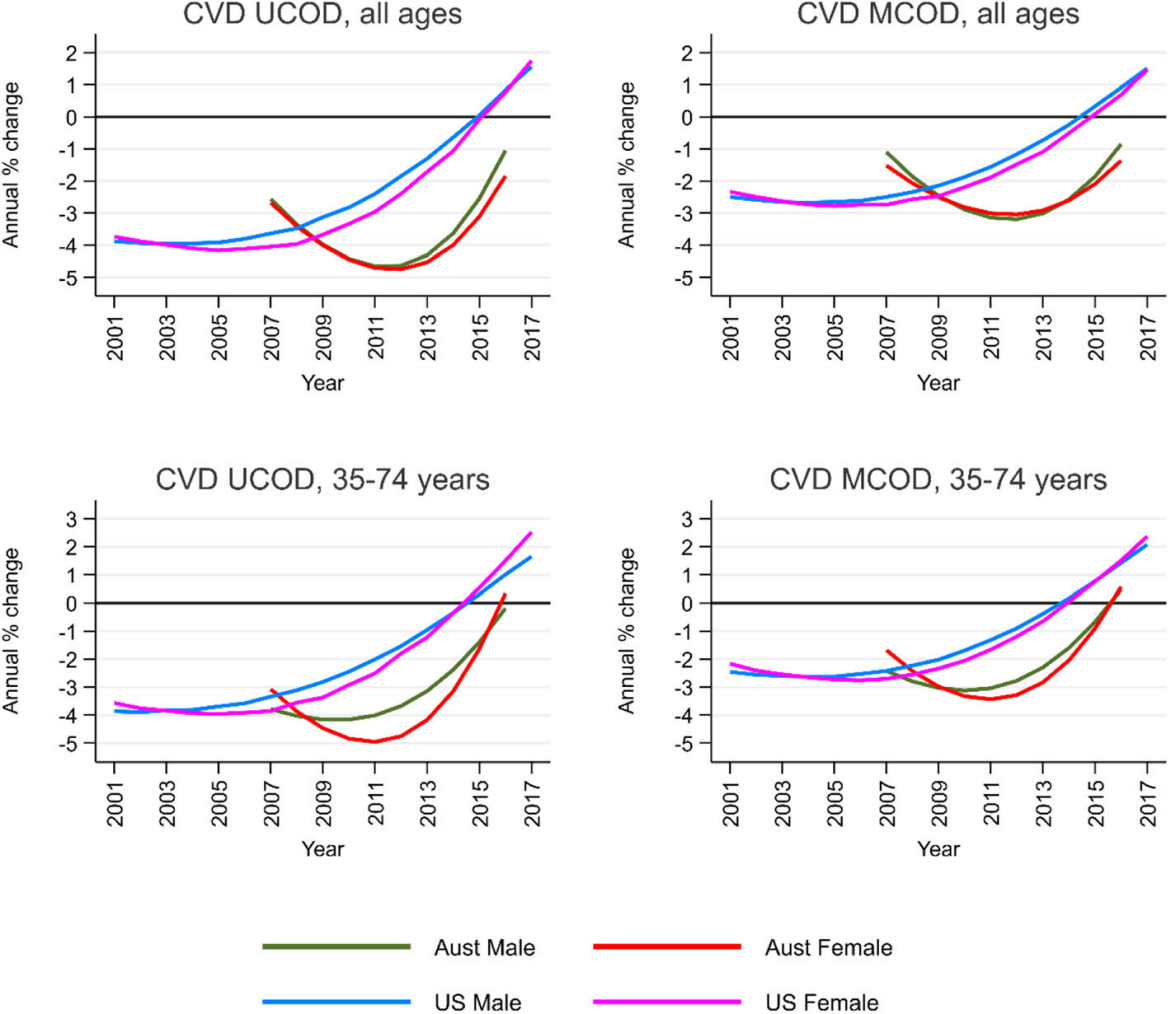
Annual change in age-standardized CVD UCOD and MCOD death rates (%), all ages, and ages 35–74 years, by sex, Australia (2007–2016) and the USA (2001–2017)

CVD UCOD and MCOD death rates in both countries declined from the beginning of the period of analysis (Additional file 5: Figure S1). However, in Australia, a slowdown in the rate of mortality decline from CVD as an UCOD and as a MCOD commenced in 2012, and in 2007 in the USA, where death rates began to increase in 2015 (Fig. 1). Notably, the rate of the slowdown in mortality decline (and then subsequent increase in the USA) occurred rapidly in each country. In Australia, male CVD UCOD and MCOD mortality rates at all ages were both declining at about 1% per annum in 2016, slightly slower than for females. In the USA, both CVD UCOD and MCOD death rates were increasing at almost 2% per annum in 2017 for both males and females. Australian and US male and female CVD death rates at ages 35–74 have performed worse than for all ages in recent years. In Australia, rates in 2016 increased slightly for CVD MCOD and were stagnant for CVD UCOD, while in the USA, both male and female CVD UCOD and MCOD were increasing at about 2% per annum in 2017.

**Fig. 2:**
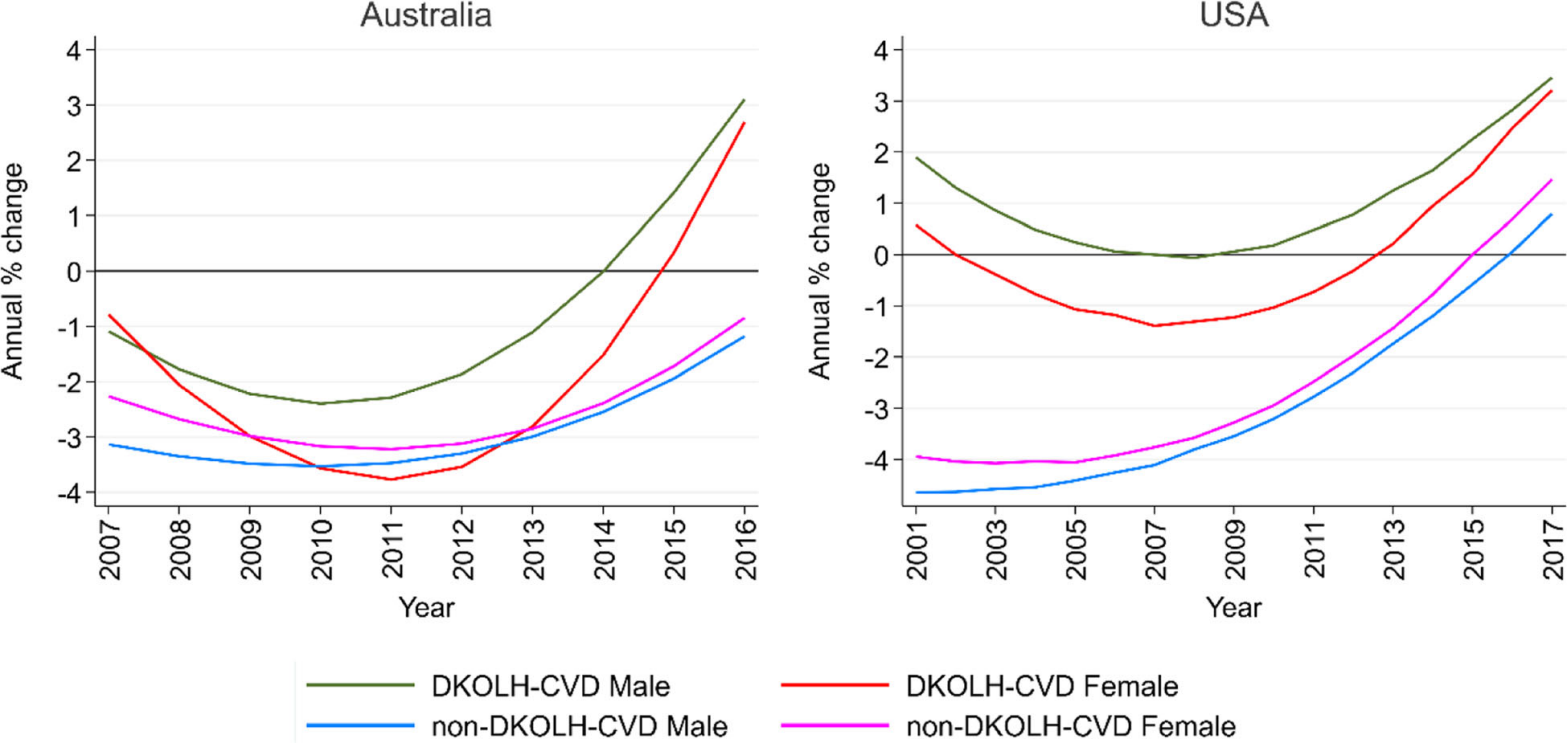
Annual change in age-standardized DKOLH-CVD and non-DKOLH-CVD CVD MCOD death rates (%), 35–74 years, by sex, Australia (2007–2016) and the USA (2001–2017)

At ages 35–74 years, Australia’s DKOLH-CVD mortality rate declined from the beginning of the study period but in more recent years has been rising at an increasingly rapid rate, reaching 3% in 2016 (Fig. 2, Additional file 6: Figure S2). US DKOLH-CVD mortality for males rose during the 2000s but for females declined until 2012. For both sexes, it was increasing at 3% per annum in 2017, although this rate of change has not risen as quickly as Australia. In both countries, non-DKOLH-CVD mortality rates were much higher than for DKOLH-CVD at the start of the period of analysis, but the gap has narrowed in Australia and fully closed in the USA because DKOLH-CVD mortality has performed worse and hence attenuated overall CVD mortality declines. Australia’s non-DKOLH-CVD mortality decline was approximately 3% per annum for much of the study period but slowed to 1% in 2016. In the USA, non-DKOLH-CVD mortality declined by at least 4% per annum in the early 2000s but was increasing for each sex in 2017. Notably, in Australia, the rate of change in DKOLH-CVD mortality has worsened more quickly than non-DKOLH-CVD mortality since CVD MCOD rate declines began slowing in 2012. Table 1 summarizes the annualized percentage change of DKOLH-CVD and non-DKOLH-CVD mortality rates at ages 35–74 years in two distinct periods.

**Table 1:** Absolute and annualized % change in age-standardized death rates (per 100,000) from specific DKOLH-CVD causes, 35–74 years, Australia (2011–2016) and the USA (2011–2017)

	Australia (2011–2016)				USA (2011–2017)			
	Male		Female		Male		Female	
	Abs. chg.	% chg.	Abs. chg.	% chg.	Abs. chg.	% chg.	Abs. chg.	% chg.
Diabetes	+ 0.4	+ 0.2	− 0.6	− 0.6	+ 3.5	+ 0.8	− 0.4	− 0.2
Hypertensive heart disease	+ 0.4	+ 0.2	− 2.1	− 1.7	+ 19.0	+ 2.6	+ 7.0	+ 1.7
Chronic kidney disease	+ 2.9	+ 4.0	+ 1.5	+ 3.4	+ 10.5	+ 6.2	+ 6.0	+ 5.6
Lipidemias	+ 1.7	+ 3.1	+ 0.6	+ 2.6	+ 2.0	+ 1.7	+ 1.1	+ 1.8
Obesity	+ 2.0	+ 5.1	+ 1.1	+ 4.2	+ 5.6	+ 5.3	+ 3.0	+ 4.1
**DKOLH-CVD**	**+ 1.2**	**+ 0.3**	**− 2.1**	**− 1.0**	**+ 21.4**	**+ 2.0**	**+ 7.9**	**+ 1.3**

These figures measure reporting of these causes on the death certificate with CVD MCOD. DKOLH-CVD does not equal to the sum of specific causes because of multiple reporting of these causes per death

*Abs. chg* absolute change, *% chg* percentage change

Analysis of changes in specific DKOLH-CVD causes shows that, in Australia, the greatest absolute and relative increase in DKOLH-CVD deaths since 2011 has been from chronic kidney disease, lipidemias, and obesity (Table 1). In contrast, both diabetes and hypertensive heart disease declined in females and increased only slightly for males. In the USA, however, the largest absolute change in DKOLH-CVD death rates since 2011 were from hypertensive diseases followed by chronic kidney disease, with comparatively large relative increases from obesity and, to a lesser extent, lipidemias. Diabetes, as for Australia, has been relatively stable since 2011.

**Fig. 3:**
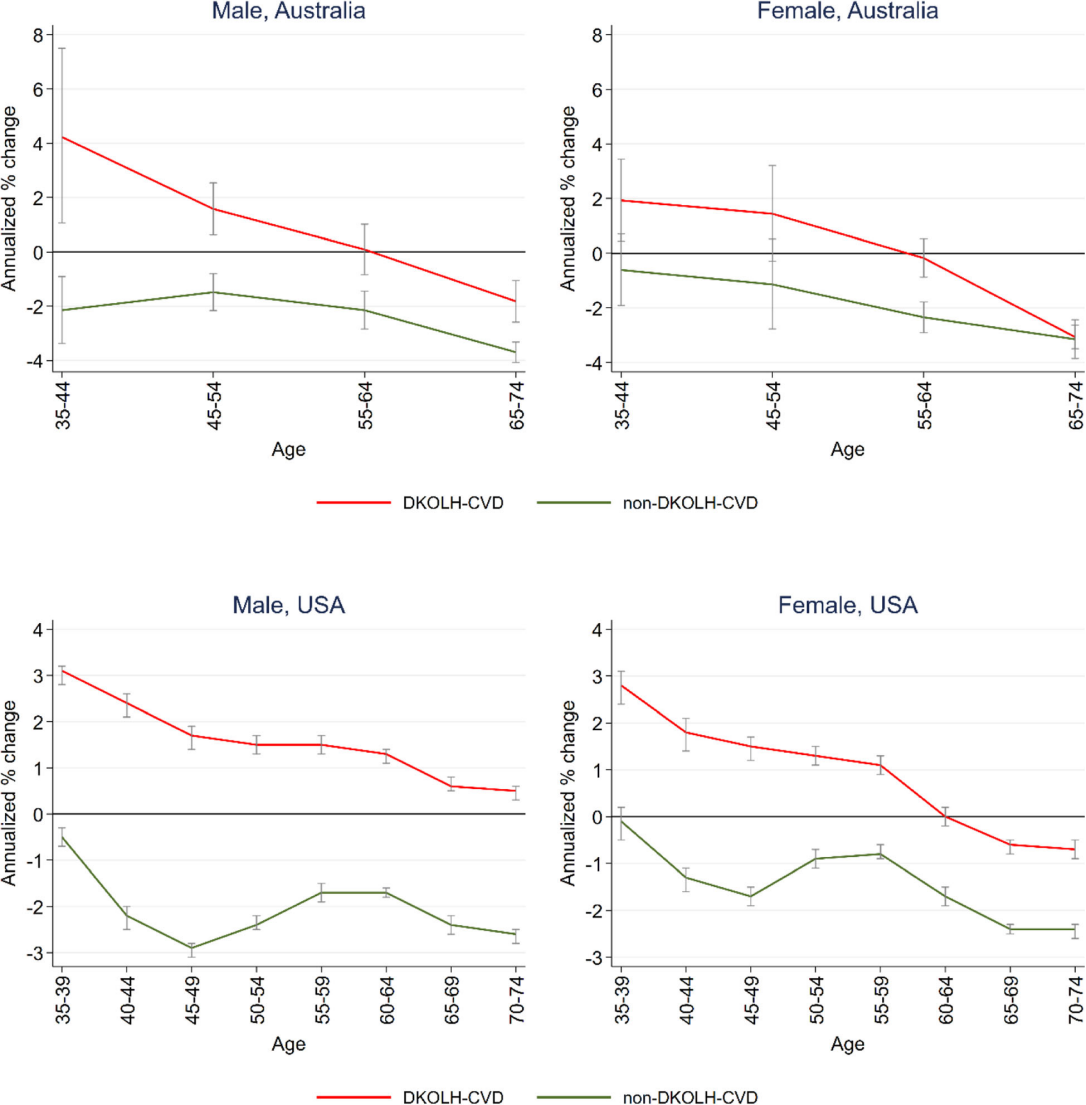
Annualized change in DKOLH-CVD and non-DKOLH-CVD MCOD age-specific death rates (% and 95% confidence intervals), by sex, Australia (2006 to 2016) and the USA (2005 to 2017). Bars indicate 95% confidence intervals

In both countries, the change in DKOLH-CVD mortality rates was worse at successively younger ages, at ages 35–44 increasing by 4% per annum among Australian males and 3% per annum for males and females at these ages in the USA (Fig. 3). The rate of decline in non-DKOLH-CVD mortality in both countries, in contrast, did not have as steep or consistent relationship with age. Although the relative change in DKOLH-CVD mortality has been worse at younger ages, these ages have lower absolute DKOLH-CVD (and non-DKOLH-CVD) mortality rates than older ages (Additional file 7: Table S5).

**Fig. 4:**
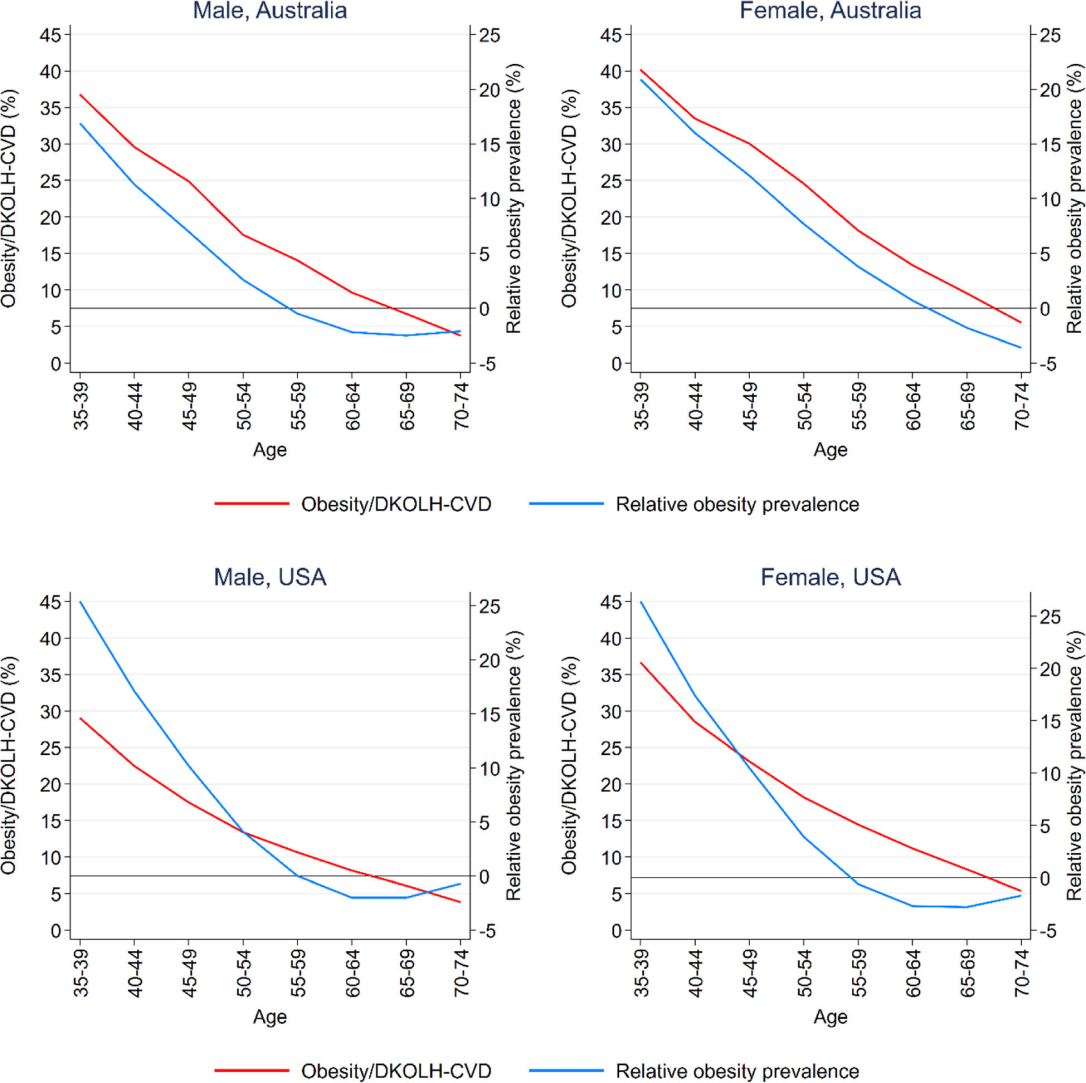
Percentage of DKOLH-CVD deaths with obesity reported, by cohort age group, and relative lifetime obesity prevalence of cohort, 1980–2015, Australia (2006–2016) and the USA (2005–2017) (%). Prev., prevalence. Age is cohort age at 2017 in the USA and 2016 in Australia

The worsening trends in DKOLH-CVD mortality at successively younger ages are consistent with two other indicators related to obesity: the percentage of DKOLH-CVD deaths with obesity reported on the death certificate and a measure of relative lifetime obesity prevalence for each cohort. In each country, there is strong consistency in the age pattern of the percentage of DKOLH-CVD deaths with reporting of obesity, ranging from between 30 and 40% at age 35–39 years and declining to only 5% at ages 70–74 years (Fig. 4). Similarly, relative obesity prevalence at ages 35–39 years in Australia is approximately 20% higher than average and in the USA about 25% higher than average, with both declining with successively older ages to be slightly below average at ages 70–74 years. Additional file 8: Figure S3 shows, at each age group, the % of DKOLH-CVD deaths due to each of the specific DKOLH-CVD causes. In each country, diabetes and lipidemias increase as a percentage of DKOLH-CVD deaths at progressively older ages, as does hypertensive heart disease in Australia but not in the USA where it remains steadily high.

## Discussion

Our study reveals that premature CVD mortality from overweight- and obesity-related causes—which we have defined as DKOLH-CVD—is rising at an increasing rate in Australia and the USA, reaching 3% per annum in recent years for both males and females, faster than non-DKOLH-CVD causes. Given that DKOLH-CVD comprises 40–50% of total CVD mortality, the finding suggests that overweight and obesity has likely been a key driver of the recent slowdown or reversal of CVD mortality decline in both countries. With CVD declines being a major factor behind life expectancy increases since the early 1970s, this finding has significant implications for public policy to extend life expectancy gains in both countries [3, 36].

In each country, DKOLH-CVD mortality increases in the past decade have been greater at progressively younger ages. In particular, the speed of DKOLH-CVD mortality increase at ages less than 45 years is alarming, notwithstanding its rise from a low base. The adverse impact of overweight and obesity at these ages is significant; these ages have a higher proportion of DKOLH-CVD mortality being reported as due to obesity and also higher cohort lifetime obesity prevalence (i.e., obesity prevalence history). The greater prominence of obesity compared with DKOLH-CVD mortality at young ages may reflect that CVD mortality is less common at younger ages and so would be more likely due to high obesity than someone with less extreme risk factors. However, the higher cohort lifetime obesity prevalence is concerning given that numerous studies have found an increased risk of mortality, including from CVD, from progressively longer duration of overweight and obesity [37–39]. Consistent with the conclusion of Preston et al., higher obesity prevalence of younger cohorts is likely to have significant adverse impact on their future mortality trends at older ages, even if it were reduced in coming years [38].

Although overweight and obesity clearly has a major role in trends in DKOLH-CVD mortality, other related risk factors such as high LDL cholesterol, high blood pressure, poor diet, and lack of exercise could well co-exist in in this cause grouping where high BMI is not present. However, annualized change in prevalence of high blood pressure since 2000, measured by the summary exposure value (a measure of risk-factor exposure adjusted for severity), has not shown a clear correlation with DKOLH-CVD mortality; in Australia, it has been steady (− 0.1% in males, + 0.2% in females) and in the USA has risen in males (+ 0.4%) and fallen in females (− 0.7%) [17]. Other CVD MCOD mortality—i.e., non-DKOLH-CVD—has also been declining at a progressively slower rate in Australia and is now rising at 1% per annum in the USA. This does suggest that risk factors other than overweight and obesity are also having an adverse influence on overall CVD mortality trends. Major non-DKOLH-CVD causes that are reported with CVD MCOD include ischemic heart disease, stroke, chronic respiratory diseases, pneumonia, specific cancers, and sepsis; it may well be that the primary risk factors for these causes are having a secondary impact on CVD. However, one such risk factor, smoking, has been declining steadily in both countries since 2000 and so would be expected to be having a downward effect on mortality [17]. This trend in non-DKOLH-CVD mortality could also be due, at least in part, to residual overweight- and obesity-related mortality not being captured by the DKOLH-CVD cluster, with excess body weight having a more pervasive impact on CVD mortality than captured by our defined set of conditions.

This study has used a novel approach of analyzing MCOD data to identify trends in overweight- and obesity-related CVD mortality. The plausibility of the DKOLH-CVD group as a proxy for overweight- and obesity-related CVD mortality can be assessed by comparing our findings with the GBD estimates of attributable fractions of CVD UCOD due to overweight and obesity (high BMI). These fractions are 34% in Australia and 41% in the USA, compared with 40% and 50%, respectively, based on our analysis (i.e., DKOLH-CVD divided by CVD MCOD). In other words, DKOLH-CVD suggests that the contribution of high BMI to CVD mortality is about 20% higher than GBD estimates [17]. While it is difficult to compare absolute contributions given the different methodological approaches (direct evidence on a group of conditions mentioned on the death certificate (DKOLH-CVD) versus the counterfactual (and hence relative) approach used in the GBD for comparative risk factor assessment), both approaches show a consistent increase in the role of overweight- and obesity-related conditions in CVD mortality trends in both countries [40].

The limitations mentioned above in the use of MCOD data to measure overweight and obesity-related CVD mortality—that DKOLH-CVD causes are likely to not fully capture the effect of overweight and obesity (but are more comprehensive that just reporting of obesity on the MCCD), that the universe of DKOLH-CVD causes is not wholly due to high BMI but would include other related risk factors, and that some non-DKOLH-CVD causes would in part be due to overweight and obesity—mean that DKOLH-CVD is more a statistically convenient amalgamation of causes known to be clinically associated with overweight and obesity than a definitive and exhaustive description of vascular diseases caused by excess body weight. Nonetheless, the PCA result that there is a stronger correlation between the individual DKOLH-CVD causes, compared with other entities reported on the death certificate along with CVD (aside from sepsis-related causes), confirms there is a common main underlying risk factor for DKOLH-CVD, namely high BMI. This PCA finding is notable given that other risk factors that lead to CVDs, such as smoking, would also be likely to have led to high component scores for certain causes (e.g., lung cancer and chronic respiratory disease), but did not. Also, the similar eigenvalues and component scores between Australia and the USA suggests quite similar CVD etiologies in the two countries, again supportive of a common risk factor of overweight and obesity. These findings are supported by the literature showing the role of overweight and obesity in increasing risk from hypertensive heart disease, diabetes, and chronic kidney disease [6, 9, 21, 22]. Reporting of mortality using MCOD data could also be biased by an increase in the number of causes reported on the death certificate; however, in neither country was this large enough to have influenced DKOLH-CVD mortality trends.

The use of MCOD has many advantages over reliance on the underlying cause of death alone. It provides direct individual-level evidence of overweight- and obesity-related mortality compared with the use of relative risks applied to CVD reported as an underlying cause to calculate population attributable fractions, as is done in the GBD, for example. Multiple cause data also better reflect the co-morbid nature of CVD, reflecting all contributory causes identified by the certifying physician rather than simply their judgment about a discrete underlying cause. Moreover, a major limitation of using underlying cause of death data for public health purposes is the frequent use of “garbage codes”, or causes which cannot or should not be used to identify the underlying cause of death. These causes have little or no public policy value and are redistributed to non-garbage codes in the GBD according to various algorithms [28]. Some causes of death, such as atherosclerosis, or within the DKOLH-CVD group, such as essential hypertension, are defined as garbage codes and are reallocated to other underlying causes, including non-CVD causes. However, when analyzed as MCODs, they can legitimately reflect the contribution of CVD or DKOLH-CVD to the death as a multiple cause and hence do not need to be redistributed to other causes.

## Conclusions

Our findings suggest that countries such as the USA and Australia are facing a public health crisis related to overweight and obesity, which has likely been a key driver the arrest of the long-term decline in CVD mortality rates in these countries. In particular, the finding that overweight and obesity are affecting CVD mortality in younger age cohorts where lifetime exposure to obesity has been much shorter than in older cohorts has significant implications for future life expectancy trends. Considering that younger cohorts are only now reaching ages where CVD mortality more commonly occurs, and that older cohorts are at ages where CVD mortality is highest, the outlook for further declines in CVD mortality in both Australia and the USA is poor, even if obesity prevalence is reduced, unless treatment improves. This, coupled with the fact that public health responses may have attenuated, but have thus far failed to significantly reduce overweight and obesity prevalence in the two countries, suggests that a much more aggressive regulatory environment, similar to what has worked for tobacco, may be required if significant further gains in life expectancy are anticipated. The lack of material impact of population-level strategies and health services efforts to reduce obesity and overweight prevalence may also reflect the markedly higher levels of obesity among the lower socioeconomic groups in high-income countries and suggests that health strategies and policies need to be more cognisant of broader structural determinants of poor health behaviors such as employment, housing, social and economic stress, and the overall management of time within families [41]. Moreover, our findings have considerable implications for other high-income countries where the CVD mortality decline is slowing, including several European countries where rising obesity has had a material impact on life expectancy in recent decades [16].

## Supplementary information

**Additional file 1: Table S1**. ICD-10 codes of causes of death and % of CVD MCOD deaths where individual cause was reported on the death certificate, by sex, Australia (2006–16) and USA (2005–17), 35–74 years.

**Additional file 2: Table S2**. Annualized % change in age-standardized death rates, DKOLH-CVD and non-DKOLH-CVD, 35–74 years, Australia 2006–11 and 2011–16, and USA 2005–11 and 2011–17.)

**Additional file 3: Table S3**. Annualized % change in age-standardized death rates, DKOLH-CVD and non-DKOLH-CVD, 35–74 years, Australia 2006–11 and 2011–16, and USA 2005–11 and 2011–17, record axis data.

**Additional file 4: Table S4**. Results from principal components analysis, Australia and USA, 35–74 years, CVD MCOD, 2005–17.

**Additional file 5: Figure S1**. Age-standardized CVD UCOD and MCOD death rates (per 100,000), all ages and 35–74 years, by sex, Australia (2006–16) and USA (2000–17).

**Additional file 6: Figure S2**. Age-standardized DKOLH-CVD and non-DKOLH-CVD CVD MCOD death rates (per 100,000), 35–74 years, by sex, Australia (2006–16) and USA (2000–17).

**Additional file 7: Table S5**. Age-specific DKOLH-CVD and non-DKOLH-CVD CVD MCOD death rates (per 100,000), 35–74 years, by sex, Australia (2016) and USA (2017).

**Additional file 8: Figure S3**. Percentage of DKOLH-CVD deaths with each specific cause reported, Australia 2006–16 and USA 2005–17, 35–74 years.

## Data Availability

The US National Center for Health Statistics Multiple Cause of Death Data files are available at https://www.cdc.gov/nchs/data_access/vitalstatsonline.htm#Mortality_Multiple. The Australian Coordinating Registry only allows access to the Australian Cause of Death Unit Record File to institutions/researchers who enter to a data access agreement (i.e., Tim Adair, Global Burden of Disease Group, University of Melbourne).

## Abbreviations

ABS: Australian Bureau of Statistics
ACR: Australian Coordinating Registry
COD URF: Cause of Death Unit Record File
CVD: Cardiovascular disease
GBD: Global Burden of Disease
DKOLH-CVD: Diabetes, chronic kidney disease, obesity, lipidemias or hypertensive heart disease, reported with cardiovascular disease
ICD-10: International Classification of Diseases Volume 10
MCCD: International Form of Medical Certificate of Cause of Death
MCOD: Multiple cause of death
NCHS: National Center for Health Statistics
NCIS: National Coronial Information System
UCOD: Underlying cause of death
