# Supplementary material for: The role of overweight and obesity in adverse cardiovascular disease mortality trends: an analysis of multiple cause of death data from Australia and the USA
Source: BMC Med. 2020 Aug 4;18:199. doi: 10.1186/s12916-020-01666-y (PMC7401233; doi:10.1186/s12916-020-01666-y)
Supplement: Supplementary file 1 — Additional file 1: Table S1. ICD-10 codes of causes of death and % of CVD MCOD deaths where individual cause was reported on the death certificate, by sex, Australia (2006–16) and USA (2005–17), 35–74 years. [file 12916_2020_1666_MOESM1_ESM.docx]

**Supplemental file: The role of overweight and obesity in adverse cardiovascular disease mortality trends: An analysis of multiple cause of death data from Australia and the United States**

**Table S1: ICD-10 codes of causes of death and % of CVD MCOD deaths where individual cause was reported on the death certificate, by sex, Australia (2006-16) and USA (2005-17), 35-74 years**

|  |  | **% of CVD MCOD deaths, 35-74 years** | | | |
| --- | --- | --- | --- | --- | --- |
| **Cause** | **ICD-10 codes** | **Australia male** | **Australia female** | **USA male** | **USA female** |
| IHD | I20-I25 | 54.6 | 37.0 | 50.3 | 38.3 |
| Other heart diseases* | I26-I51 | 38.1 | 42.3 | 38.3 | 41.1 |
| Stroke | I60-I69 | 17.4 | 23.5 | 13.3 | 17.6 |
| Hypertensive heart disease | I10-I13 | 20.6 | 23.5 | 31.8 | 32.4 |
| Other CVD | I00-I09, I14-I19, I52-I59, I70-I99 | 13.7 | 15.5 | 10.8 | 11.8 |
| Diabetes | E10-E14 | 18.3 | 18.9 | 19.6 | 21.4 |
| Chronic kidney | N18 | 6.7 | 7.9 | 6.9 | 8.2 |
| Lipidemias | E78 | 4.7 | 4.4 | 5.1 | 4.5 |
| Obesity | E65-E66 | 3.3 | 4.6 | 4.1 | 5.4 |
| Other endocrine, nutritional and metabolic diseases | D66-D99, E00-E09, E15-E64, E67-E77, E79-E99 | 4.3 | 6.7 | 3.9 | 6.1 |
| Alcohol-related diseases** | F10, K70, K74, K76 (excl. K76.3), X45 | 7.5 | 4.2 | 7.1 | 3.8 |
| Pneumonia | J18 | 6.0 | 6.5 | 4.2 | 4.7 |
| Chronic respiratory | J40-J44 | 11.7 | 13.1 | 11.9 | 14.1 |
| Other respiratory diseases | J00-J17, J19-J39, J45-J99 | 12.7 | 16.1 | 11.1 | 14.5 |
| Nervous system diseases | G00-G29, G32-G99 | 7.6 | 8.1 | 6.1 | 7.0 |
| Dementia | F00-F03, G30-G31 | 2.8 | 3.8 | 1.9 | 2.7 |
| Digestive diseases | K00-K69, K71-K73, K75, K76.3, K77, K99 | 7.5 | 8.3 | 5.1 | 5.7 |
| Lung cancer | C33-C34 | 5.6 | 5.7 | 3.5 | 3.7 |
| Prostate cancer | C61 | 2.5 | - | 1.1 | - |
| Breast cancer | C50 | 0.0 | 4.9 | 0.0 | 2.4 |
| Colorectal cancer | C18-C21 | 2.1 | 1.9 | 1.0 | 1.0 |
| Other cancers | C00-C17, C22-C32, C35-C49, C51-C60, C62-D49 | 15.3 | 17.0 | 7.4 | 8.2 |
| Other genitourinary diseases | N00-N16, N20-N99 | 2.5 | 3.4 | 2.1 | 2.9 |
| Other kidney diseases | N17, N19 | 5.6 | 6.6 | 5.3 | 5.9 |
| Sepsis | A41 | 4.9 | 6.1 | 5.0 | 6.6 |
| Other infections | A00-A40, A42-B99 | 2.5 | 2.3 | 3.1 | 2.7 |
| Injuries | V00-X44, X46-Y99 | 8.3 | 8.1 | 5.9 | 5.4 |
| DKOLH-CVD*** | - | 39.6 | 40.9 | 49.8 | 49.7 |

* Excludes I46 (cardiac arrest), unless it is the UCOD.

** Includes alcoholic liver disease, fibrosis and cirrhosis of liver, mental and behavioural disorders due to use of alcohol, accidental poisoning by and exposure to alcohol.

*** Figure for 2016 in Australia and 2017 in the US.
