# Supplementary material for: The role of overweight and obesity in adverse cardiovascular disease mortality trends: an analysis of multiple cause of death data from Australia and the USA
Source: BMC Med. 2020 Aug 4;18:199. doi: 10.1186/s12916-020-01666-y (PMC7401233; doi:10.1186/s12916-020-01666-y)
Supplement: Supplementary file 2 — Additional file 2: Table S2. Annualized % change in age-standardized death rates, DKOLH-CVD and non-DKOLH-CVD, 35–74 years, Australia 2006–11 and 2011–16, and USA 2005–11 and 2011–17.) [file 12916_2020_1666_MOESM2_ESM.docx]

**Additional File 2**

**Table S2: Annualized % change in age-standardized death rates, DKOLH-CVD and non-DKOLH-CVD, 35-74 years, Australia 2006-11 and 2011-16, and USA 2005-11 and 2011-17**

| **Australia** | **Male** | | | **Female** | | |
| --- | --- | --- | --- | --- | --- | --- |
| **DKOLH-CVD MCOD** | **% change** | **95% CI (Lower, Upper)** | **% change** | | **95% CI (Lower, Upper)** |  |
| 2006-11 | -2.0 | -2.6, -1.3 | -2.6 | | -3.1, -2.1 |  |
| 2011-16 | +0.3 | -0.4, +1.0 | -1.0 | | -1.5, -0.4 |  |
| **Non-DKOLH-CVD MCOD** |  |  |  | |  |  |
| 2006-11 | -3.4 | -4.1, -2.7 | -2.9 | | -3.7, -2.0 |  |
| 2011-16 | -2.4 | -3.2, -1.6 | -2.2 | | -3.1, -1.2 |  |
| **USA** | **Male** | | | **Female** | | |
| **DKOLH-CVD MCOD** | **% change** | **95% CI (Lower, Upper)** | **% change** | | **95% CI (Lower, Upper)** |  |
| 2005-11 | +0.1 | -0.1, +0.3 | -1.2 | | -1.3, -1.0 |  |
| 2011-17 | +2.0 | +1.8, +2.3 | +1.3 | | +1.1, +1.6 |  |
| **Non-DKOLH-CVD MCOD** |  |  |  | |  |  |
| 2005-11 | -3.6 | -3.8, -3.5 | -3.3 | | -3.5, -3.2 |  |
| 2011-17 | -0.8 | -1.1, -0.6 | -0.3 | | -0.6, -0.1 |  |

CI: Confidence interval
