# Supplementary material for: The role of overweight and obesity in adverse cardiovascular disease mortality trends: an analysis of multiple cause of death data from Australia and the USA
Source: BMC Med. 2020 Aug 4;18:199. doi: 10.1186/s12916-020-01666-y (PMC7401233; doi:10.1186/s12916-020-01666-y)
Supplement: Supplementary file 4 — Additional file 4: Table S4. Results from principal components analysis, Australia and USA, 35–74 years, CVD MCOD, 2005–17. [file 12916_2020_1666_MOESM4_ESM.docx]

**Additional File 4**

**Table S4: Results from principal components analysis, Australia and USA, 35-74 years, CVD MCOD, 2005-17**

|  | **Australia** | | **USA** | |
| --- | --- | --- | --- | --- |
|  | **Component 1** | **Component 2** | **Component 1** | **Component 2** |
| **Eigenvalue** | **1.6331** | **1.5588** | **1.7856** | **1.5278** |
| **Hypertensive heart** | -0.0711 | **0.4185** | -0.1649 | **0.5467** |
| **Diabetes** | -0.0136 | **0.5238** | -0.0854 | **0.4988** |
| **Chronic kidney** | 0.1613 | **0.3429** | 0.0838 | **0.2339** |
| **Lipidemias** | -0.1314 | **0.3790** | -0.1529 | **0.3855** |
| **Obesity** | -0.0139 | **0.2440** | -0.0670 | **0.2212** |
| IHD | -0.4106 | 0.2202 | -0.3837 | -0.0417 |
| Other heart | 0.2502 | -0.0051 | 0.2652 | -0.2338 |
| Stroke | 0.0079 | -0.1834 | 0.0501 | 0.0016 |
| Other CVD | 0.2301 | -0.0375 | 0.2127 | 0.0903 |
| Other endocrine | 0.2808 | 0.0734 | 0.2546 | 0.1564 |
| Alcohol-related | 0.2350 | -0.0010 | 0.1415 | 0.0919 |
| Other respiratory | 0.1829 | -0.0534 | 0.3206 | -0.0220 |
| Pneumonia | 0.1512 | -0.0671 | 0.2619 | -0.0053 |
| Chronic respiratory | 0.0460 | -0.0342 | 0.0671 | -0.0039 |
| Nervous system | 0.0438 | 0.0507 | 0.0775 | 0.1034 |
| Dementia | -0.0044 | -0.0163 | 0.0160 | 0.0799 |
| Digestive | 0.3514 | 0.0568 | 0.2793 | 0.1347 |
| Lung cancer | -0.0112 | -0.1535 | 0.0519 | -0.0684 |
| Prostate cancer | -0.0003 | 0.0010 | 0.0051 | 0.0395 |
| Breast cancer | 0.0553 | -0.0627 | 0.0394 | 0.0192 |
| Colorectal cancer | 0.0449 | -0.0058 | 0.0368 | 0.0375 |
| Other cancers | 0.1430 | -0.1165 | 0.1232 | 0.0452 |
| Other genitourinary | 0.1833 | 0.1740 | 0.1856 | 0.1516 |
| Other kidney | 0.2972 | 0.2043 | 0.2459 | 0.0858 |
| Sepsis | 0.3513 | 0.1120 | 0.3837 | 0.1012 |
| Injuries | 0.1292 | -0.0156 | 0.0357 | 0.0034 |
| Other infections | 0.2479 | 0.0167 | 0.2369 | 0.1273 |
