# Supplementary material for: The role of overweight and obesity in adverse cardiovascular disease mortality trends: an analysis of multiple cause of death data from Australia and the USA
Source: BMC Med. 2020 Aug 4;18:199. doi: 10.1186/s12916-020-01666-y (PMC7401233; doi:10.1186/s12916-020-01666-y)
Supplement: Supplementary file 5 — Additional file 5: Figure S1. Age-standardized CVD UCOD and MCOD death rates (per 100,000), all ages and 35–74 years, by sex, Australia (2006–16) and USA (2000–17). [file 12916_2020_1666_MOESM5_ESM.docx]

**Additional File 5**

**Figure S1: Age-standardized CVD UCOD and MCOD death rates (per 100,000), all ages and 35-74 years, by sex, Australia (2006-16) and USA (2000-17)**


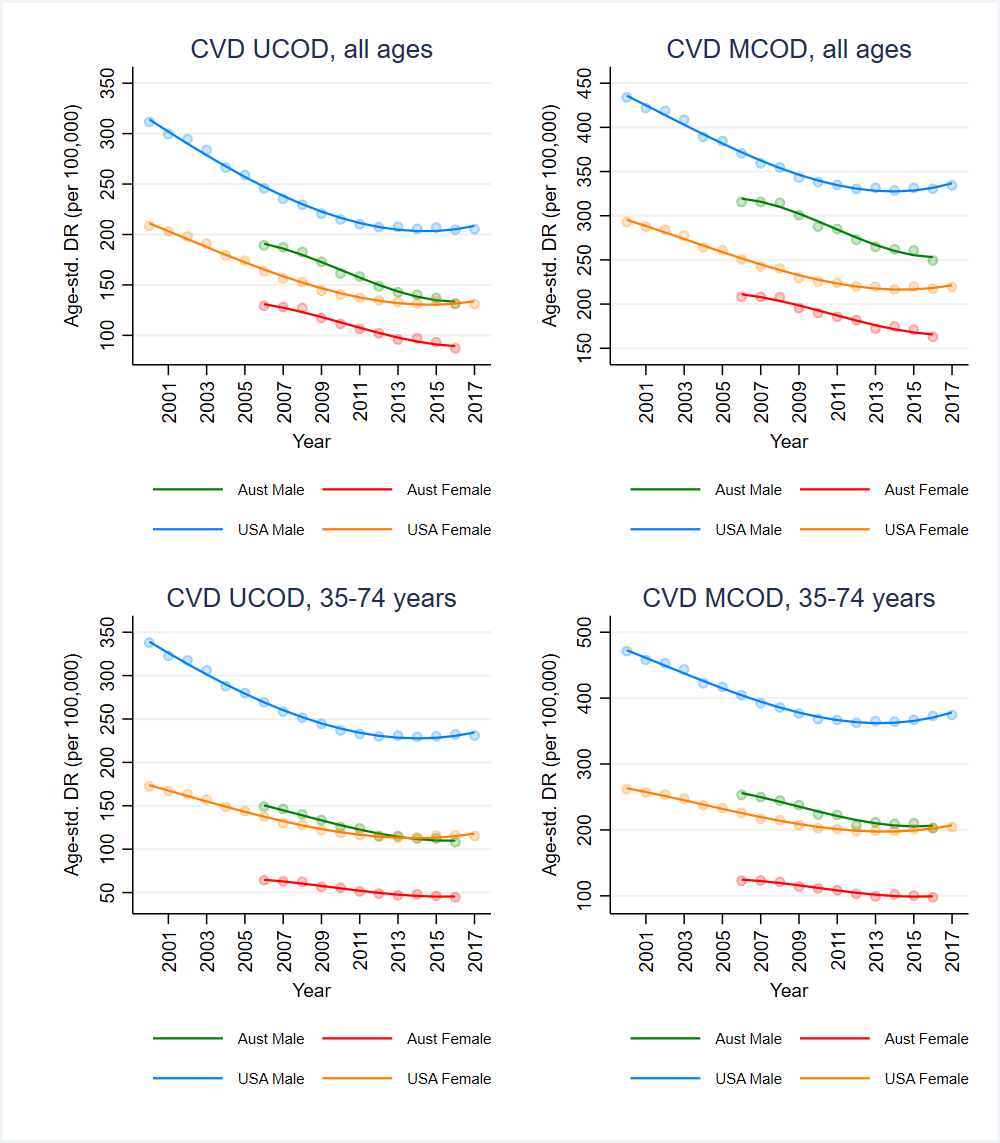


Lines are smoothed rates. Dots are un-smoothed rates. DR: Death rates
