# Supplementary material for: The role of overweight and obesity in adverse cardiovascular disease mortality trends: an analysis of multiple cause of death data from Australia and the USA
Source: BMC Med. 2020 Aug 4;18:199. doi: 10.1186/s12916-020-01666-y (PMC7401233; doi:10.1186/s12916-020-01666-y)
Supplement: Supplementary file 7 — Additional file 7: Table S5. Age-specific DKOLH-CVD and non-DKOLH-CVD CVD MCOD death rates (per 100,000), 35–74 years, by sex, Australia (2016) and USA (2017). [file 12916_2020_1666_MOESM7_ESM.docx]

**Additional File 7**

**Table S5: Age-specific DKOLH-CVD and non-DKOLH-CVD MCOD death rates (per 100,000), 35-74 years, by sex, Australia (2016) and USA (2017)**

| **Australia** | | | | | **USA** | | | | |
| --- | --- | --- | --- | --- | --- | --- | --- | --- | --- |
| **Age group** | **Male** | | **Female** | | **Age group** | **Male** | | **Female** | |
|  | **DKOLH-CVD** | **Non-DKOLH-CVD** | **DKOLH-CVD** | **Non-DKOLH-CVD** |  | **DKOLH-CVD** | **Non-DKOLH-CVD** | **DKOLH-CVD** | **Non-DKOLH-CVD** |
| **35-44** | 10 | 27 | 5 | 13 | **35-39** | 26 | 30 | 13 | 17 |
| **45-54** | 34 | 69 | 14 | 31 | **40-44** | 47 | 46 | 24 | 26 |
| **55-64** | 100 | 157 | 48 | 63 | **45-49** | 81 | 73 | 41 | 40 |
| **65-74** | 303 | 396 | 158 | 204 | **50-54** | 136 | 127 | 69 | 66 |
|  |  |  |  |  | **55-59** | 217 | 205 | 112 | 105 |
|  |  |  |  |  | **60-64** | 327 | 323 | 168 | 164 |
|  |  |  |  |  | **65-69** | 460 | 468 | 255 | 254 |
|  |  |  |  |  | **70-74** | 689 | 757 | 423 | 454 |
