# Supplementary material for: The role of overweight and obesity in adverse cardiovascular disease mortality trends: an analysis of multiple cause of death data from Australia and the USA
Source: BMC Med. 2020 Aug 4;18:199. doi: 10.1186/s12916-020-01666-y (PMC7401233; doi:10.1186/s12916-020-01666-y)
Supplement: Supplementary file 8 — Additional file 8: Figure S3. Percentage of DKOLH-CVD deaths with each specific cause reported, Australia 2006–16 and USA 2005–17, 35–74 years. [file 12916_2020_1666_MOESM8_ESM.docx]

**Additional File 8**

**Figure S3: Percentage of DKOLH-CVD deaths with each specific cause reported, Australia 2006-16 and USA 2005-17, 35-74 years**


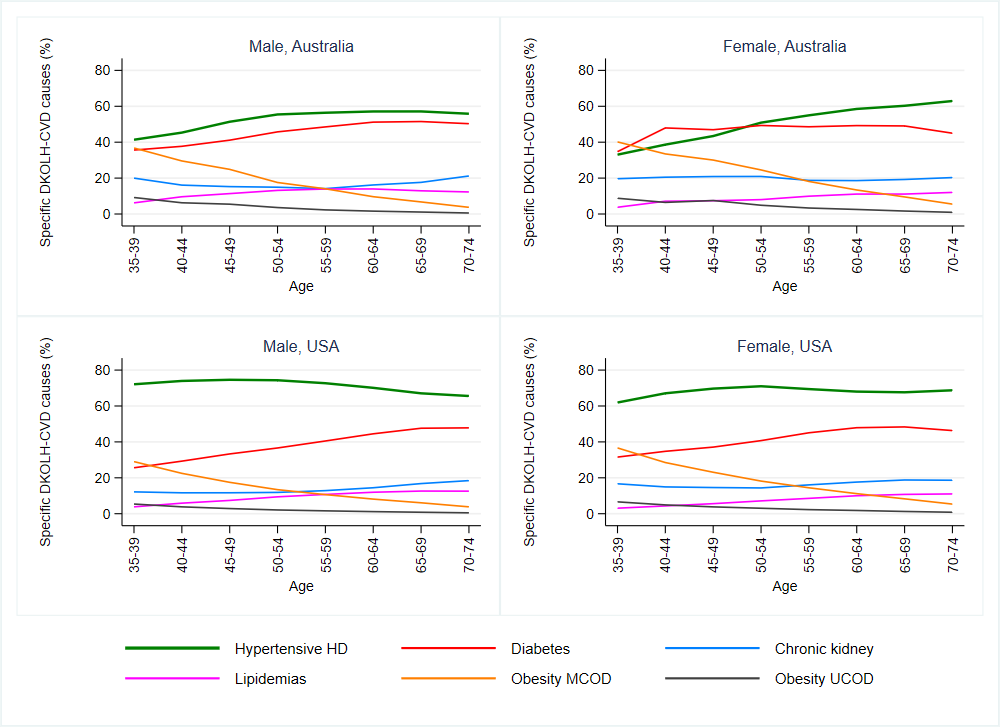


HD: Heart disease
